# Dyschloremia Is a Risk Factor for the Development of Acute Kidney Injury in Critically Ill Patients

**DOI:** 10.1371/journal.pone.0160322

**Published:** 2016-08-04

**Authors:** Min Shao, Guangxi Li, Kumar Sarvottam, Shengyu Wang, Charat Thongprayoon, Yue Dong, Ognjen Gajic, Kianoush Kashani

**Affiliations:** 1 Division of Nephrology and Hypertension, Department of Medicine, Mayo Clinic, Rochester, MN, United States of America; 2 Multidisciplinary Epidemiology and Translational Research in Intensive Care (METRIC) Research Group, Mayo Clinic, Rochester, MN, United States of America; 3 Department of Critical Care Medicine, Anhui Provincial hospital Affiliated to Anhui Medical University, Hefei, Anhui, China; 4 Department of Pulmonary Medicine, Guang’Anmen Hospital, China Academy of Chinese Medical Sciences, Beijing, China; 5 Department of Pulmonary Medicine, The First Affiliated Hospital of Xi’an Medical University, Shaanxi, China; 6 Division of Pulmonary and Critical Care Medicine, Department of Medicine, Mayo Clinic, Rochester, MN, United States of America; Mario Negri Institute for Pharmacological Research and Azienda Ospedaliera Ospedali Riuniti di Bergamo, ITALY

## Abstract

**Introduction:**

Dyschloremia is common in critically ill patients, although its impact has not been well studied. We investigated the epidemiology of dyschloremia and its associations with the incidence of acute kidney injury and other intensive care unit outcomes.

**Material and Methods:**

This is a single-center, retrospective cohort study at Mayo Clinic Hospital—Rochester. All adult patients admitted to intensive care units from January 1^st^, 2006, through December 30^th^, 2012 were included. Patients with known acute kidney injury and chronic kidney disease stage 5 before intensive care unit admission were excluded. We evaluated the association of dyschloremia with ICU outcomes, after adjustments for the effect of age, gender, Charlson comorbidity index and severity of illness score.

**Results:**

A total of 6,025 patients were enrolled in the final analysis following the implementation of eligibility criteria. From the cohort, 1,970 patients (33%) developed acute kidney injury. Of the total patients enrolled, 4,174 had a baseline serum chloride. In this group, 1,530 (37%) had hypochloremia, and 257 (6%) were hyperchloremic. The incidence of acute kidney injury was higher in hypochloremic and hyperchloremic patients compared to those with a normal serum chloride level (43% vs.30% and 34% vs. 30%, respectively; *P* < .001). Baseline serum chloride was lower in the acute kidney injury group vs. the non-acute kidney injury group [100 mmol/L (96–104) vs. 102 mmol/L (98–105), *P* < .0001]. In a multivariable logistic regression model, baseline serum chloride of ≤94 mmol/L found to be independently associated with the risk of acute kidney injury (OR 1.7, 95% CI 1.1–2.6; *P* = .01).

**Discussion:**

Dyschloremia is common in critically ill patients, and severe hypochloremia is independently associated with an increased risk of development of acute kidney injury.

## Introduction

Acute kidney injury (AKI) is a grave and common complication of critical illness. Despite significant progress in the care of critically ill patients, the mortality rate in AKI patients remains high. Recent studies indicate AKI incidence among all hospital admissions is 3–10%, in-the general hospital wards, and intensive care unit (ICU) mortality rates are 20% and 50%, respectively [1, 2]. Annually, about two million patients die of AKI [3] and those who survive AKI are apt to develop chronic kidney disease (CKD) [4, 5].

Appropriate AKI risk stratification among ICU patients is helpful to prevent AKI and its progression and/or design trials to test the utilization of therapeutic options. Knowing each individual risk profile is critical in the process of preventive and/or therapeutic interventions. Sepsis, trauma, shock, nephrotoxic agents, and contrast media exposure are known risk factors for AKI. Despite growing knowledge in the field, there are several other risk factors that have not been well described or identified.

Chloride is one of most affluent anions in the plasma and interstitial fluid. It accounts for approximately one-third of plasma tonicity and participates in acid-base balance [6]. Serum sodium serves as the primary extracellular cation and serum chloride as the primary extracellular anion [7]. Several studies have examined the epidemiology of sodium disturbances and their possible impact on adverse outcomes in critically ill patients [8–10]. The incidence of dysnatremia in ICU patients varies between 25% and 45%. Even mild hyponatremia and hypernatremia is associated with significantly higher mortality and longer duration of hospitalization [10]. Although chloride abnormalities, particularly hypochloremia, are very common in critical care settings, they have not received appropriate attention.

In comparison with the volume of literature in dysnatremia, the number of studies reporting the incidence and impact of dyschloremia on patient outcomes is very limited. Hypochloremia is associated with metabolic alkalosis. Infusing chloride-rich solutions like normal saline may be the first choice for the resuscitation of patients with alkalemia and hypochloremia. On the other hand, a growing volume of evidence indicates the use of chloride-rich intravascular fluids are associated with high occurrence of AKI, metabolic acidosis, and hyperkalemia. This association is more evident when these solutions are administrated in large quantities [11–14].

Despite significant progress in the field, the current body of knowledge on the incidence and impact of baseline plasma chloride on clinical outcomes, and on AKI specifically, are very preliminary. This study aimed to explore the association of baseline serum chloride and the development of AKI in ICU patients. We hypothesized that baseline serum chloride is closely associated with AKI development during ICU stay.

## Materials and Methods

### Patients and study design

**Fig 1:**
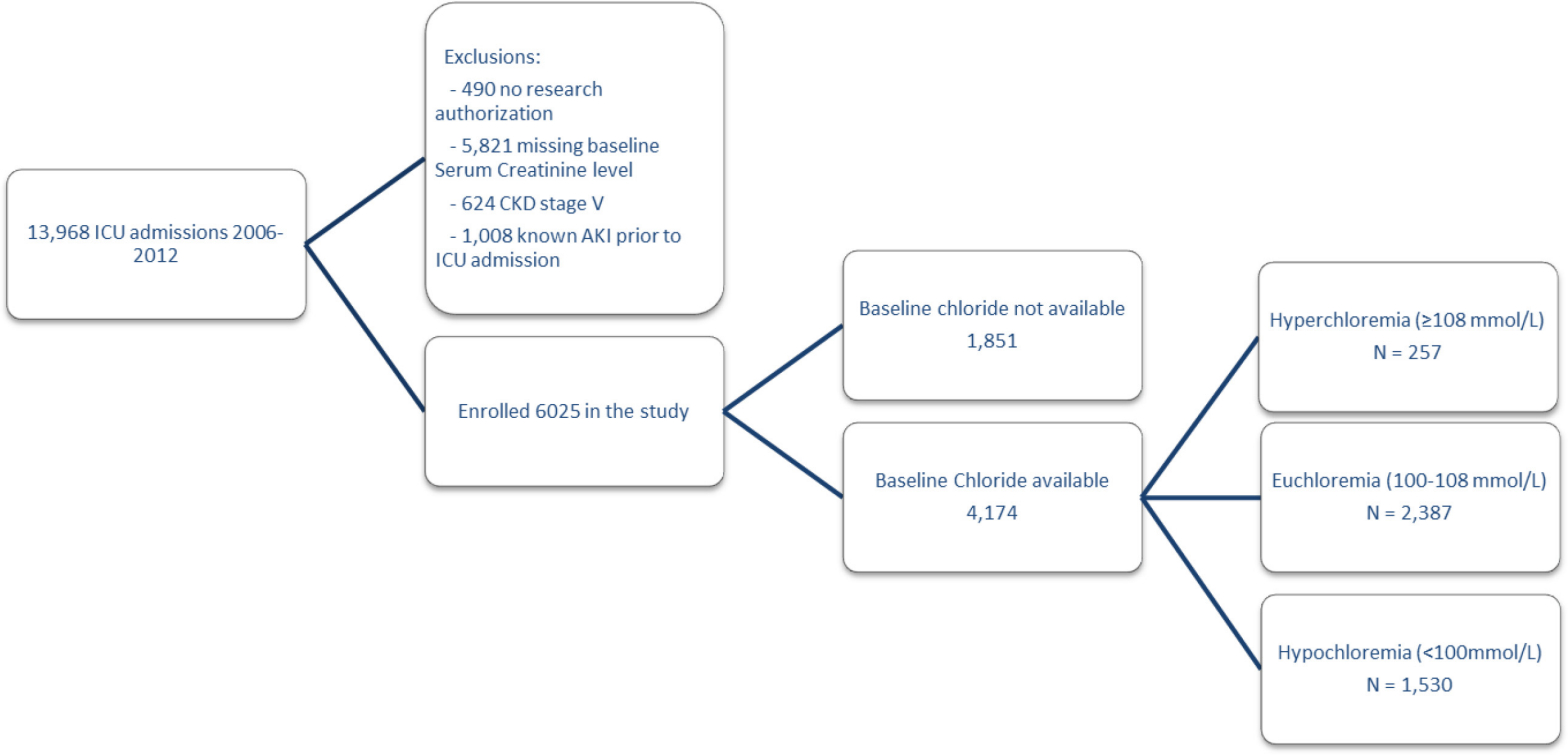
Study design and a flowchart of the recruitment process and the reasons for exclusion. Abbreviations: AKI, acute kidney injury; CKD, chronic kidney disease.

This study is a historical cohort investigation of all adult individuals who resided in Olmsted County, Minnesota, and were admitted to the ICU. We screened 13,968 patients who were admitted to the ICU at Mayo Clinic Hospital—Rochester from January 1, 2006, through December 30, 2012. All the information was abstracted from the electronic health records (EHR). A large dataset of ICU patients from January 1^st,^ 2006 through December 31^st^, 2012, was built. In this database, we used a validated AKI sniffer to detect AKI according to the Kidney Disease Improving Global Outcomes (KDIGO) criteria. We excluded patients with missing baseline serum creatinine, or outcome data or and those who lack research authorization. We also excluded pre-existing end-stage kidney disease (ESKD) or known AKI prior to admission according to the ICD codes. For patients with more than one episode of ICU admission, all the parameters of each admission were counted separately. Among all patients who met the inclusion criteria (Olmsted County residents older than 18 years), those with available baseline serum chloride were included in the final analysis (Fig 1). The Mayo Clinic Institutional Review Board approved the study and waived written consent for those who provided research authorization.

### Data collection

We abstracted the data from the EHR. The databases we used have been described in previous studies. They included, “Mayo Clinic Hospital admissions and diagnoses among Olmsted County residents”, and we also used Advanced Cohort Explorer (ACE) and ICU Data Mart. The ACE is a clinical data repository that contains patient demographics, diagnoses, laboratory, flow sheets, clinical notes, and pathology reports with the ability to search via text based queries. ICU Data Mart is a Microsoft Structured query language (SQL) database.

The Query Language (MS SQL) (Microsoft Corporation, Redmond, WA) data warehouse has the ability to abstract all pertinent clinical data from the EHR on a near real-time basis. Within Data Mart, all the static data including the Minnesota death registry are updated quarterly. [15, 16] For the adjudication of AKI, we used a validated automated electronic tool (AKI sniffer) [17]. Baseline demographic parameters and the baseline blood chemistry panel (including serum creatinine, sodium, bicarbonate, chloride, and other electrolytes) measured during the month prior to the index ICU admission, were recorded. If patients have several results of chloride as well as other electrolytes in the baseline period, we chose the first results so as to exclude the effect of the medical intervention on baseline lab tests. The Acute Physiology and Chronic Health Evaluation (APACHE) III and Sequential Organ Failure Assessment (SOFA) scores were abstracted. Using the EHR, we calculated Charlson’s comorbidity index (CCI) for risk adjustment [18]. Other clinical information, including laboratory data, the volume of infused normal saline, ICU and hospital mortality, and length of stay (LOS) were obtained from the computerized databases. To evaluate the relationship between baseline serum chloride and the incidence of AKI, we recorded serum chloride values during the month prior to the index ICU admission. The serum chloride levels were measured by an automated chemistry analyzer in the central hospital laboratory. The reference range for this chloride assay was 100–108 mmol/L. The patients were classified into four groups according to the baseline serum chloride levels: ≤94 and 95–99 mmol/L groups (severe and mild hypochloremia; n = 1,530), 100–108 mmol/L (euchloremic; n = 2,387), and >108 mmol/L (hyperchloremic; n = 257).

### Outcome variables and associated comorbidities

The primary objective of the study was to evaluate the incidence of dyschloremia and its relationship with the development of AKI following ICU admission. Diagnosis of ICU-acquired AKI was determined by using the Kidney Disease Improving Global Outcomes (KDIGO) criteria [11]. Secondary outcomes included the LOS in the ICU and hospital, and ICU and in-hospital mortality.

### Statistical analysis

Continuous variables were summarized as mean±standard deviation (SD) or median and interquartile range (IQR), based on the normality of distribution. Categorical variables were reported as count and percentages. Continuous variables were compared with an independent samples *t*-test or the Mann-Whitney *U* test. Categorical variables were compared using the chi-square test or Fisher’s exact tests. Logistic regression was used to identify variables that predict the onset and progression of AKI and other secondary outcomes. Statistical analysis was carried out using JMP (Volume 10.0, SAS Institute Inc., NC, USA) software. A two-sided *P* value < .05 was considered to indicate statistical significance.

## Results

During the study period, 13,968 adults were admitted to an ICU at Mayo Clinic Hospital—Rochester, of which 6,025 (43.1%) met the eligibility criteria. Of these, 4,078 patients were admitted to the medical ICUs and 1,947 to the surgical ICU. We identified 4,174 patients who had baseline serum chloride available and included them in the outcome analysis based on the baseline serum chloride level (Fig 1). In the enrolled cohort 6,025 patients, 50% (n = 3,207) individuals were male, and the mean (±SD) age was 62.9 (±19.6) years. The median (IQR) of CCI, APACHE III, and SOFA scores were 2 (0–4), 48 (33–65), and 3(1–6), respectively. The 0.9% saline infusion was used in 5,526 (92%) of individuals with the median amount of 1,300 (625–2,900) mL throughout ICU admission. AKI was detected in 1,970 (32.7%) patients, of which 1,083 (18%) were in stage I, 507 (8.4%) in stage II, and 380 (6.3%) in stage III. Overall ICU mortality was 3.6% (n = 216), and in-hospital mortality was 7.2% (n = 432). The ICU and hospital LOS were 1.1 (0.8–2.2) and 4.9 (2.6–8.4) days, respectively. In the cohort with available baseline serum chloride, the median (IQR) baseline serum chloride was 101 (97–104) mmol/L, which increased to 103 (100–107) mmol/L, during ICU stay.

### Baseline Dyschloremia

**Fig 2:**
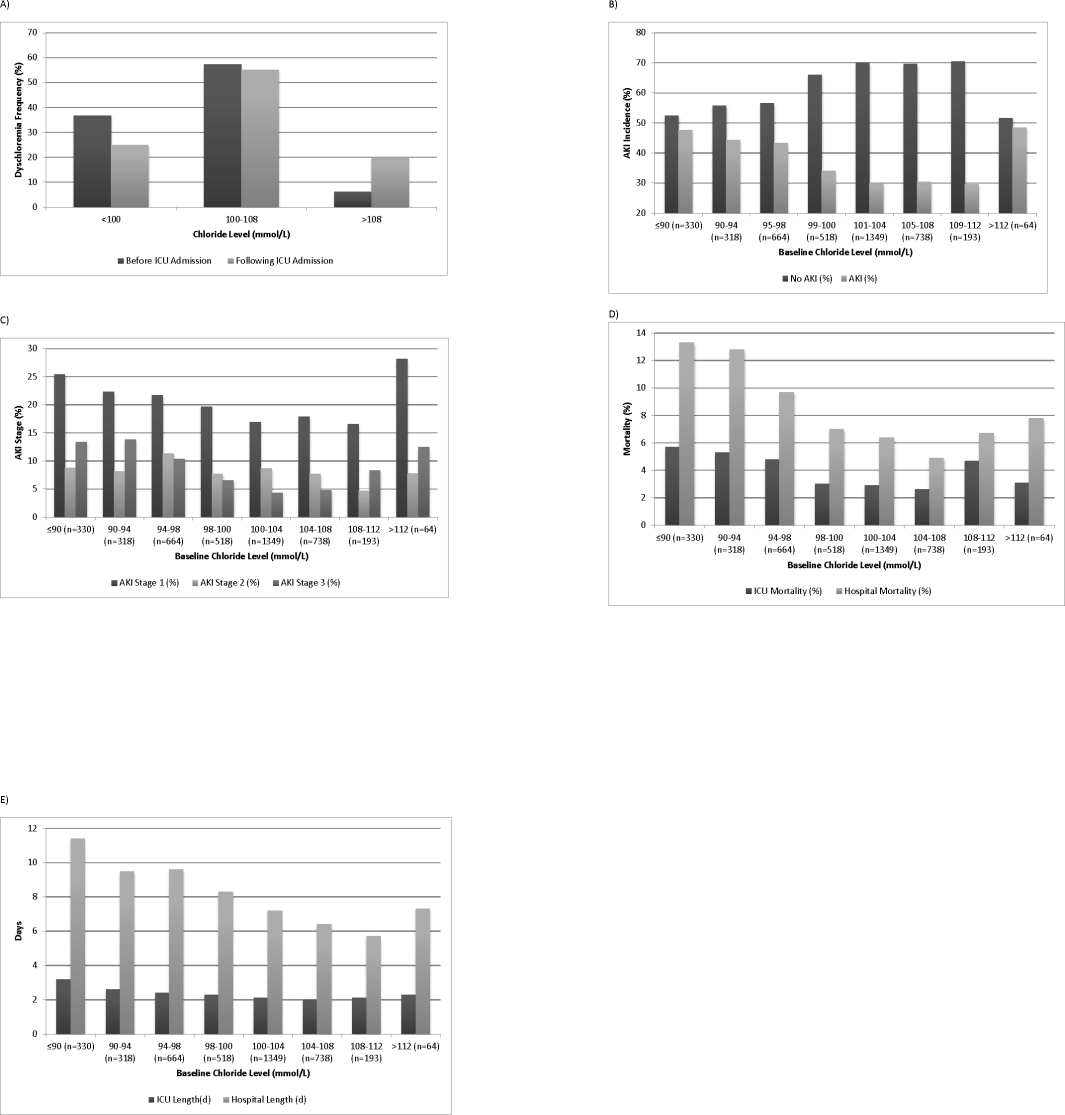
A) Frequency of dyschloremia before and after the ICU admission; B) Incidence of AKI according to the baseline serum chloride concentrations; C) Incidence of AKI stages according to the baseline serum chloride concentrations; D) Hospital and intensive care unit (*ICU*) mortality according to different baseline serum chloride concentrations; E) Hospital and ICU length of stay (LOS) according to different baseline serum chloride concentrations.

Within the cohort with available baseline serum chloride (N = 4,174), the prevalence of hypochloremia and hyperchloremia prior to the index ICU admission were 37% (n = 1,530) and 6% (n = 257), respectively (Fig 2A). Following ICU admission, 25% (n = 1,039) and 20% (n = 835) of patients became hypochloremic and hyperchloremic, respectively (Fig 2A).

Patients with hyperchloremia at baseline were younger (59±22 years vs. 63±20 years, *P =* .005) and had fewer comorbid conditions than patients with baseline euchloremia (*P* = .02). Hypochloremic patients, when compared with euchloremic patients, were older (67±18 vs. 63±20, *P* < .001), had a higher prevalence of comorbid conditions (3[1–5] vs. 1[0–4], *P* < .001), lower levels of sodium (135[131–138] vs.138 [136–140], P < .0001), and higher levels of bicarbonate (25[22–28] vs. 25[22–27], P < .0001).They also had higher severity of illness scores and a prevalence of CKD than euchloremic and hyperchloremic patients (Table 1).

**Table 1:** Characteristics and Outcome of Patients with Baseline Chloride Disturbance^*^.

Variable	Baseline Serum Chloride					
	Hyperchloremia (A) ≥108mmol/L (N = 257)	Euchloremia (B) 100-108mmol/L (N = 2,387)	Hypochloremia (C) <100mmol/L(N = 1,530)	*P* value (A vs. B)	*P* value (B vs. C)	*P* value (A vs. C)
ICU type, n (%)				0.0004	< .0001	0.15
Medical	193(75.1)	1526(63.9)	1082(70.7)			
Surgical	64(24.9)	861(36.1)	448(29.3)			
**Demographic characteristics**						
Age, years	59±22	63±20	67±18	0.005	< .0001	< .0001
Male	134 (52.1)	1,296 (54.3)	750 (49.0)	0.51	0.0013	0.35
**Comorbidities and severity of illnesses, n (%)**						
ACS	30 (11.7)	368 (15.4)	288 (18.8)	0.1	0.005	0.006
CHF	20 (7.8)	283 (11.9)	322 (21.1)	0.05	< .0001	< .0001
PVD	12 (4.7)	160 (6.7)	154 (10.1)	0.2	0.0002	0.006
CVA	35 (13.6)	315 (13.2)	248 (16.2)	0.9	0.009	0.3
COPD	15 (5.8) ^c^	342 (14.3)	367 (24.0)	0.0002	< .0001	< .0001
Diabetes	48 (18.7)	574 (24.1)	522 (34.1)	0.05	< .0001	< .0001
DM with complication	12(4.7)	165(6.9)	209(13.7)	0.2	< .0001	< .0001
Cirrhosis	4 (1.6)	75 (3.1)	73(4.8)	0.2	0.009	0.02
CKD	54 (21.0)	404 (16.9)	82 (25.0)	0.1	< .0001	0.2
Peptic ulcer disease	22 (8.6)	187 (7.8)	186 (12.2)	0.7	< .0001	0.09
Severe liver disease	1 (0.4)	28 (1.20)	27 (1.8)	0.3	0.1	0.1
Tumor	45 (17.5)	509 (21.3)	423 (27.8)	0.2	< .0001	0.0006
Charlson score	1 (0–4)	1 (0–4)	3 (1–5)	0.08	< .0001	< .0001
APACHEIII	49 (29–65)	47 (31–64)	55 (41–72)	0.03	< .0001	0.007
SOFA	4 (2–6)	3 (1–6)	4 (2–6)	0.0001	< .0001	0.46
**Outcomes**						
AKI	88 (34.2)	726 (30.4)	664 (43.3)	0.2	< .001	0.006
AKI stage I	50 (19.5)	423 (17.7)	338 (22.1)	0.5	0.0007	0.3
AKI stage II	14 (5.5)	198 (8.3)	146 (9.5)	0.1	0.2	0.03
AKI stage III	24 (9.3)c	105 (4.4)	180 (11.8)	0.0005	< .001	0.3
Use of MV	87 (33.9)	817 (34.2)	553 (36.1)	0.9	0.2	0.5
ICU length of stay, days	1.1 (0.9–2.1)	1.2 (0.9–2.2)	1.5 (0.9–2.8)	0.9	0.0008	0.03
ICU mortality, n (%)	11 (4.3)	70 (2.9)	73 (4.8)	0.2	0.003	0.7
Hospital length of stay, days	3.9 (1.9–7.7)	4.9 (2.7–8.3)	6.5 (3.7–11.6)	0.002	< .001	< .0001
Hospital mortality, n (%)	18 (7)	146 (6.1)	161 (10.5)	0.6	< .0001	0.08
**Lab test**						
Baseline Scr available	**n = 254**	**n = 2,368**	**n = 1,441**			
Baseline Scr, mg/dL	1.1 (0.8–1.4)	0.9 (0.8–1.2)	0.9 (0.7–1.3)	0.0003	< .0001	0.55
Serum albumin available	**n = 46**	**n = 432**	**n = 505**			
Serum albumin, g/dL	3.3 (2.7–3.7)	3.4 (2.9–3.8)	3.2 (2.7–3.7)	0.6	0.01	0.6
Baseline Cl min (mmol/L)	111 (109–113)	103 (101–105)	95 (91–98)	< .0001	< .0001	< .0001
Baseline Na (mmol/L)	141(139–143)	138(136–140)	135(131–138)	< .0001	< .0001	< .0001
Baseline Bicarbonate(mmol/L)	22(19–24)	25(22–27)	25(22–28)	< .0001	0.2	< .0001
**Fluid administration**						
Data available	**n = 236**	**n = 2,144**	**n = 1,420**			
0.9% Saline (mL)	1375 (812–3100)	1250 (600–2550)	2000 (1000–3819)	0.08	< .0001	0.009
Diuretic use, (%)	56 (22)	812 (34)	720 (46)	< .0001	< .0001	< .0001

**Abbreviations:** ACS; acute coronary syndrome, AKI; acute kidney injury, APACHE III; Acute Physiology and Chronic Health Evaluation score, CHF; chronic heart failure, CKD; chronic kidney disease, COPD; chronic obstructive pulmonary disease, CVA; cerebrovascular accident, MV; mechanical ventilation, ICU; intensive care unit, PVD; peripheral vascular disease, Scr; serum creatinine, SOFA; Sequential Organ Failure Assessment score

*Continuous variables are expressed as mean (±SD) or median and interquartile range (IQR); categorical variables are expressed as frequency (n) and percentage (%).

The baseline serum chloride was inversely correlated with SOFA (r = -0.005, *P* = .14), APACHE III (r = -0.08, *P* = .002), and CCI (r = -0.012, *P* < .001) scores, and serum bicarbonate level (r = -0.14, *P* < .0001) but had a direct relation to the serum sodium level (r = 0.81, *P* < .0001). Patients with a baseline hypochloremia compared to euchloremic patients received a 0.9% saline infusion (2000 [1000–3819] vs. 1250 [600–2550], *P* < .001). In a multivariable logistic regression analysis, age (OR 1.003, 95% CI 1.001–1.01; *P* = .3), congestive heart failure (OR 1.6, 95% CI 1.28–2.01; *P* < .0001), CKD (OR 1.4, 95% CI 1.1–1.7; *P* = .002), dementia (OR 1.6, 95% CI 1.1–2.3; *P* = .02), chronic obstructive pulmonary disease(COPD) (OR 1.6, 95% CI 1.4–2.0; *P* < .0001), diabetes mellitus (DM) (OR 1.3, 95% CI 1.0–1.5; *P* = .02), DM with complication (OR 1.4, 95% CI 1.1–1.9; *P* = .02), peptic ulcer disease (OR 1.4, 95% CI 1.1–1.8; *P* = .01), and metastatic cancer (OR 2.7, 95% CI 1.9–3.8; *P* < .0001) were associated with a greater risk of baseline hypochloremia. Moreover, use of diuretics was associated with lower baseline serum chloride (OR 1.4, 95% CI 1.2–1.7; *P* < .001), sodium (OR 0.75, 95% CI 0.73–0.76; *P* < .0001), and bicarbonate (OR 1.08, 95% CI 1.06–1.09; *P* < .0001) levels (Table 2).

**Table 2:** Factors associated with Hypochloremia. Univariate and Multivariate Analysis.

Variable(n = 4,174)	Univariate Analysis		Multivariate Analysis	
	Odds Ratio 95% CI	*P* value	Odds Ratio 95% CI	*P* value
Age	1.01 (1.01–1.02)	< .001	1.003 (1.0–1.01)	.3
Male	0.82 (0.72–0.93)	.002	0.82 (0.70–0.95)	.01
CHF	2.06 (1.74–2.46)	< .001	1.61 (1.28–2.01)	< .0001
CKD	1.59(1.36–1.85)	< .001	1.40 (1.13–1.72)	.002
COPD	2.02 (1.72–2.38)	< .001	1.65 (1.35–2.02)	< .0001
Rheumatologic diseases	1.80 (1.38–2.36)	< .001	1.34 (0.96–1.86)	.09
Dementia	1.49 (1.10–2.05)	.01	1.57 (1.07–2.33)	.02
Diabetes mellitus	1.68 (1.47–1.93)	< .001	1.26 (1.03–1.53)	.02
Peptic ulcer disease	1.61 (1.31–1.99)	< .001	1.42 (1.09–1.84)	.008
Cirrhosis	1.63 (1.17–2.25)	.004	1.27 (0.84–1.90)	0.3
DM with complication	2.21 (1.79–2.73)	< .001	1.44 (1.06–1.94)	0.02
Metastatic cancer	2.90 (1.60–2.74)	< .001	2.71 (1.96–3.76)	< .0001
Diuretics	1.77 (1.56–2.02)	< .001	1.42 (1.20–1.68)	< .0001
Serum Sodium	0.78(0.76–0.79)	.0001	0.75(0.73–0.76)	< .0001
Serum Bicarbonate	1.02(1.01–1.04)	.0007	1.08(1.06–1.09)	< .0001

**Abbreviations:** CHF; chronic heart failure **AKI and ICU outcomes.**

During the course of the study, 1,970 (32.7%) out of 6,025 patients developed AKI while in the ICU. Dialysis-requiring AKI was seen in 61 (9.4%), 69 (5.8%), 40 (1.9%), and 7 (2.7%) of patients with serum chloride levels ≤94, 94–99, 100–108, >108 mmol/L, respectively. When compared with individuals who did not develop AKI, patients with AKI were older (67.4±18.3 years vs. 60.7±19.8 years; *P* < .001), had a higher CCI (2[1–5] vs. 1[0–3]; *P* < .001), were more likely to be on mechanical ventilation (48.8% vs. 27.4%, *P*<0.001), had higher hospital mortality (14.9% vs. 3.4%; *P* < .001), and longer hospital (7 [4–12] vs. 4 [2–7]; P < .001) and ICU (2 [1–4] vs. 1 [1–2]; *P* < .001) LOS. Patients with AKI received a larger volume of 0.9% saline infusion than non-AKI patients (2100 [1000–4000] vs. 1101 [500–2250] mL; *P* < .001), and they also had significantly lower baseline serum chloride (100 [96–104] vs. 102 [98–105]; *P* < .001), baseline serum sodium (138 [134–140] vs. 138[135–140]; P = 0.0013); and baseline serum bicarbonate lab results (24 [21–27] vs. 25[22–27]; *P* < .0001) (S1 Table).

In comparison with euchloremic and hyperchloremic patients, there was a higher incidence of AKI in patients who were hypochloremic at the baseline (43.3% in hypochloremics vs. 30.4% in euchloremics vs. 34.2% in hyperchloremics; *P* < .001). When they were compared with euchloremic patients, they also had higher ICU (4.8% vs. 2.9%) and hospital (10.5% vs. 6.1%) mortality rates (*P* < .001) and ICU (1.5 [1–3] vs. 1 [1–2] days) and hospital (7 [4–12] vs. 5 [3–8] days) LOS (*P* < .001) (Table 1).

In subgroup of patients with baseline hyperchloremia, the incidence of AKI, ICU, and hospital mortality in patients without acidosis in comparison with those with acidosis was not different [(33.3% vs. 35.3%; *P* = .0.74); (4.4% vs. 4.1%, *P* = 0.89); (5.9% vs. 8.2%, *P* = 0.47), respectively]. The ICU and hospital LOS was not different between the two groups [(1.2 vs. 1.1 days; *P* = 0.22); (3.9 vs. 3.9 days; *P* = 0.59), respectively] (S2 Table).

In a logistic regression analysis, age (OR 1.01, 95% CI 1.001–1.02; *P* = .04), SOFA Score (OR 1.31, 95% CI 1.24–1.38; *P* < .0001), baseline serum creatinine (OR 1.30, 95% CI 1.14–1.52; *P* = .001), baseline serum albumin <3.5g/L (OR 1.56, 95% CI 1.12–2.17; *P* = .01), baseline bicarbonate (OR 0.96, 95% CI 0.92–0.99; *P* = 0.01), baseline serum chloride ≤94 mmol/L (OR 1.72, 95% CI, 1.07–2.79; *P* = .02), diuretics (OR 1.7, 95% CI 1.2–2.3; *P* = .0.002) were independently associated with a greater risk of AKI (Table 3). S3 Table describes the results of univariate and multivariate analysis for in-hospital mortality.

**Table 3:** Factors associated with AKI. Univariate and Multivariate Analysis.

Variable (n = 4,174)	Univariate Analysis		Multivariate Analysis	
	Odds Ratio 95% CI	*P* value	Odds Ratio 95% CI	*P* value
Age, y	1.02 (1.01–1.021)	< .001	1.01 (1.001–1.02)	.04
Male	0.90 (0.80–0.997)	.04	0.88 (0.64–1.20)	.4
CHF	2.29 (1.97–2.66)	< .001	1.29 (0.81–2.05)	.3
CKD	2.69 (2.35–3.08)	< .001	2.05 (1.21–3.48)	.008
COPD	1.27 (1.10–1.46)	.001	1.40 (0.92–2.13)	.1
Cirrhosis	1.36(1.02–1.82)	.04	1.39(0.72–2.70)	.3
Dementia	1.58(1.22–2.05)	.0007	1.22(0.55–2.73)	.6
Diabetes mellitus (DM)	1.76(1.56–1.98)	< .001	1.05(0.69–1.59)	.8
DM with complication	1.70(1.41–2.04)	< .001	1.29(0.66–2.54)	.5
Metastatic Cancer	1.24(0.97–1.58)	.09	1.95(0.72–5.32)	.2
Peptic ulcer disease	1.17(0.96–1.40)	.11	1.34(0.80–2.27)	.3
Rheumatologic disease	1.05(0.82–1.34)	.72	1.36(0.74–2.52)	.3
SOFA Score	1.32 (1.30–1.35)	< .001	1.31 (1.24–1.38)	< .0001
Charlson comorbidity Index	1.12 (1.10–1.14)	< .001	1.09 (0.97–1.23)	.2
Baseline Sodium	0.98(0.97–0.99)	< .001	0.99(0.96–1.03)	.8
Baseline Bicarbonate	0.95(0.84–0.96)	< .001	0.96(0.92–0.99)	.01
Baseline Scr, mg/dL	1.99 (1.82–2.20)	< .001	1.30 (1.14–1.52)	.001
Baseline albumin <3.5 g/L	2.01 (1.60–2.53)	< .001	1.56 (1.12–2.17)	.01
Baseline chloride, ≤94 mmol/L	1.69 (1.43–2.01)	< .001	1.72 (1.07–2.79)	.02
Baseline chloride, 94–99 mmol/L	1.26 (1.10–1.45)	.001	1.25 (0.87–1.81)	.2
Baseline chloride, 100–108 mmol/L	1.00 (reference)	-	1.00 (reference)	--
Baseline chloride, >108 mmol/L	1.06 (0.81–1.38)	.7	1.01 (0.46–2.18)	.9
Diuretics	2.00 (1.78–2.25)	< .001	1.67(1.20–2.32)	.002
0.9% Saline ≥1550 mL	2.81 (2.50–3.15)	< .001	1.71(1.22–2.40)	.002

**Abbreviations:** CHF, chronic heart failure; CKD, chronic kidney disease; COPD, chronic obstructive pulmonary disease; Scr, serum creatinine; SOFA, Sequential Organ Failure Assessment score.

The dose-response relationship between different categories of baseline serum chloride and risk of AKI in our patients followed a U-shaped curve. We found both low and high serum chloride values associated with the increased incidence of AKI (Fig 2B and 2C), ICU and hospital mortality (Fig 2D), and the LOS (Fig 2E).

## Discussion

This retrospective study is the first report to evaluate the incidence of dyschloremia in the ICU and demonstrates the association of low baseline serum chloride with AKI. We also showed that hypochloremic patients had a tendency to have higher hospital mortality and a longer LOS in both the ICU and hospital.

Dyschloremia in critically ill patients is very common and appear to be associated with worse outcomes [19, 20]. One study showed that the prevalence of chloride abnormalities in the critical care setting was 25% (hypochloremia 9% and hyperchloremia 17%)[21]. The authors also reported that hypochloremic patients tend to have a worse outcome. Another report noted that 32% of the ICU patients had hyperchloremia, with a prevalence that was approximately 1.9 times greater than the rate observed in the previously mentioned report[22]. In our investigation, baseline hypochloremia was seen in 37% of patients which decreased to 25% following ICU admission. Hyperchloremia was noted in 6% of patients prior to ICU admission and increased to 20% in the ICU. These changes in chloride levels could be attributed to the use of chloride-rich fluids for intravascular volume expansion.

The most important causes of hypochloremia in critically ill patients were regarded to be increased loss or decreased intake of chloride. For example, loss of gastric fluid, the use of diuretics or adrenal insufficiency, water toxicity, and malnutrition could lead to hypochloremia. In our patients, we found that chronic conditions such as COPD, DM, dementia, metastatic cancers, and the use of diuretics were associated with the hypochloremia even after adjustments for age and gender. We also showed that the baseline serum chloride was inversely correlated with severity of illness and comorbid conditions.

The clinical significance of baseline serum chloride derangements on the ICU outcomes has not been extensively evaluated. Few studies have reported hypochloremia to be associated with the increased likelihood of poor outcomes. In patients with chronic obstructive pulmonary disease exacerbation, Terzano et al. showed patients with hypochloremia have a longer duration of non-invasive mechanical ventilation [23]. In addition,Tani et al., showed that in critically ill patients, hypochloremia led to longer ICU and hospital stays and higher mortality [21].

In our study, patients with baseline hypochloremia had a longer length of ICU and hospital stay compared to patients without hypochloremia. Fig 2B and 2C show that serum chloride level of ≤94 mmol/L was significantly associated with a higher incidence of AKI. The relationship between serum chloride levels and mortality also follows the same U-shaped relationship. In multivariate logistic regression analysis, we found hypochloremia, along with other factors such as age, CKD, baseline serum creatinine, baseline bicarbonate, and serum albumin levels, are independent risk factors for AKI. We were not able to show that high a chloride level at baseline is associated with an increased risk of AKI and hospital mortality. In our cohort, the number of patients with elevated chloride at the baseline is very small (257 out of 4,174 patients; 6%). Thus, our cohort is most likely underpowered to show a significant impact of baseline hyperchloremia on the risk of AKI.

As the relationship between low baseline hypochloremia and risk of AKI has not been described before, there is no characterization of the mechanism of such a relationship presented in the literature. We hypothesized that the relationship between baseline hypochloremia and AKI was mainly due to its association with potential dehydration due to decreased nutritional and fluid intake in the setting of multiple chronic comorbidities. Our investigation is not able to confirm any causality relationship between hypochloremia and AKI. This is mainly due to its retrospective nature. Critically ill patients who have multiple comorbidities often suffer from poor homeostasis. Unfortunately, we do not have data regarding patient volume status prior to admission. Therefore, we have included serum sodium in the analysis as a potential surrogate for volume shifts. We found a direct positive relationship between sodium and chloride level. Thus, it is conceivable that the etiology of AKI in patients with hypochloremia was most likely hypovolemia and not necessarily hypochloremia itself. Furthermore, a negative relationship between hypochloremia and severity of illness scores may indicate that baseline hypochloremia could be used as a prognostic indicator for ICU patients.

In contrary to the association of baseline hypochloremia and AKI, the relationship between in-hospital hyperchloremia and AKI is well described. Some *in vitro* and animal studies have shown that high serum chloride can result in afferent arteriolar vasoconstriction, decreased renal artery blood flow, and reduced glomerular filtration rates, which leads to salt and water retention [24, 25]. A study of 109,836 patients with systemic inflammatory response syndrome (SIRS) demonstrated that a fluid resuscitation strategy using lower chloride loads was associated with lower in-hospital mortality [26]. Another study of 30,994 adult patients undergoing major open abdominal surgery showed that patients who received 0.9% saline had a higher unadjusted in-hospital mortality rate when compared with patients treated with balanced crystalloids [11]. In a landmark study [12], Yunos et al., found that a chloride-restrictive strategy for fluid resuscitation is associated with a lower incidence of AKI. A post hoc analysis revealed a significant decrease in the use of renal replacement therapy in the chloride-restricted arm.

None of the above-mentioned studies reported the relationship between baseline serum chloride levels and AKI, however. In our study, we noted that critically ill patients with low baseline chloride received higher doses of chloride-rich solutions than those with normal or high serum chloride levels at the baseline (2000 mL [1000–3819] in hypochloremia group vs. 1250 mL [600–2550] in normal chloride group and 1375 mL [812–3100] in hyperchloremic patients; *P* < .001). We also found administration of 0.9% saline in excess of 1550 ml is an independent risk factor of AKI. One can hypothesize that patients with hypochloremia at the baseline are more volume depleted; therefore, they may have a higher risk for AKI. In these patients, receiving additional normal saline could serve as the second hit which increases the chance of AKI, per sé. Our findings are opposite to the discoveries of the Saline vs Plasma-Lyte 148 (PL-148) for ICU fluid Therapy (SPLIT) trial [27]. In this study, authors did not find any difference between chloride-rich and balanced solutions among the participants. In the SPLIT trial, the average amount of fluids received was 2000mL[1000-3500mL] for buffered crystalloid vs. 2000 mL [1000–3250 mL] for the chlorid-rich arm. Future trials including larger sample size with the higher amount of fluid intake may be necessary to address this issue.

As with other retrospective studies, our report is subject to several limitations. First, this study was a single-center study, and most of the patients who participated in the study were Caucasian. This may limit the generalizability of our conclusions. Second, our cohort includes a highly selected sample of critically ill patients who had baseline chloride level available (<30% of 13,968 screened patients). Third, our retrospective analysis to find a relationship between chloride level disturbances and AKI is based on the historical dataset and is subject to biases related to such analytic designs. Fourth, a significant number of patients had missing data regarding their volume balance. Therefore, despite being aware of limitations associated with this surrogacy, we used serum sodium as a surrogate for the volume status.

Future prospective studies are warranted to clarify whether baseline hypochloremia is just a biomarker or plays a pathophysiologic role in the course of AKI. Given the limitations of our study, a multiple-center, large cohort study with an intervention arm to correct hypochloremia is necessary to validate our findings.

## Conclusions

Serum chloride level derangements are common in critically ill patients, and severe hypochloremia is independently associated with an increased risk of ICU and hospital mortality, LOS, and AKI. Prospective randomized trials are needed to validate our findings and evaluate the impact of chloride normalization in patients’ outcomes.

## Supporting Information

S1 Table Multivariable Analysis of In-Hospital Mortality. (DOCX)

S2 Table Comparison of Variables in Non-Acute Kidney Injury and Acute Kidney Injury Patients in the Intensive Care Unit. (DOCX)

S3 Table Multivariable Analysis of In-Hospital Mortality. (DOCX)
