# Supplementary material for: Dyschloremia Is a Risk Factor for the Development of Acute Kidney Injury in Critically Ill Patients
Source: PLoS One. 2016 Aug 4;11(8):e0160322. doi: 10.1371/journal.pone.0160322 (PMC4974002; doi:10.1371/journal.pone.0160322)
Supplement: S1 Table — (DOCX) [file pone.0160322.s001.docx]

**Supplementary Table 1:** Comparison of Variables in Non-Acute Kidney Injury and Acute Kidney Injury Patients in the Intensive Care Unit ^a^

| Variable | Non-AKI | AKI | *P* value |
| --- | --- | --- | --- |
|  | N=4,055 | N=1,970 |  |
| **Demographic characteristics** | | | |
| Age*,* years | 60.7$\pm$19.8 | 67.4$\pm$18.3 | <.001 |
| Male | 2195 (54.1) | 1012 (51.4) | .04 |
| **ICU admission, n (%)** |  |  |  |
| CCU &MICU | 2825(69.7) | 1253(63.6) | <.0001 |
| SICU | 1230(30.3) | 717(36.4) |  |
| **Comorbidities, n (%)** | | | |
| ACS | 574 (14.2) | 344 (17.5) | .001 |
| CHF | 409 (10.1) | 402 (20.4) | <.001 |
| PVD | 253 (6.24) | 177 (9.0) | .001 |
| Dementia | 138 (3.4) | 104 (5.3) | .001 |
| CVA | 475 (11.7) | 314 (15.9) | <.001 |
| COPD | 604 (14.9) | 358 (18.2) | .001 |
| Diabetes | 907 (22.4) | 663 (33.7) | <.001 |
| Cirrhosis | 122 (3.0) | 80 (4.1) | .03 |
| Chronic kidney disease | 517 (12.8) | 556 (28.2) | <.001 |
| Severe liver disease | 12 (8.1) | 43 (18.5) | .005 |
| Tumor | 858 (21.2) | 477 (24.2) | .01 |
| Lymphoma | 96 (2.4) | 65 (3.3) | .03 |
| **Score** | | | |
| CCI | 1 (0-3) | 2 (1-5 ) | <.001 |
| APACHE III | 40 (28-54) | 63 (48-82) | <.001 |
| SOFA | 2 (1-4) | 5 (3-8) | <.001 |
| **Outcomes** | | | |
| Use of MV | 1110 (27.4) | 962 (48.8) | <.001 |
| ICU LOS, days | 1.0 (0.8-1.7) | 1.9 (1.0-3.8) | <.001 |
| ICU mortality | 56 (1.4) | 160 (8.1) | <.001 |
| Hospital LOS, days | 4.0 (2.2-7.0) | 6.9 (4.1-11.7) | <.001 |
| Hospital mortality | 138 (3.4) | 294 (14.9) | <.001 |
| **Laboratory tests** | | | |
| **Baseline Scr** | **n=3,294** | **n=1,627** |  |
| Baseline Scr, mg/dL | 0.9 (0.7-1.1) | 1.1 (0.8-1.6) | <.001 |
| **Baseline albumin** | **n=662** | **n=584** |  |
| Baseline albumin, g/dL | 3.9 (3.5-4.3) | 3.6 (3.2-4) | <.001 |
| **Baseline Chloride** | **n=2,696** | **n=1,478** |  |
| Baseline Chloride, mmol/L | 102 (98-105) | 100 (96-104) | <.001 |
| Baseline Sodium, mmol/L | 138 (135-140) | 138(134-140) | 0.0013 |
| Baseline bicarbonate, mmol/l | 25(22-27) | 24(21-27) | <.0001 |
| **Fluid and diuretics** | **n=3,655** | **n=1,871** |  |
| Saline 0.9% administration, mL | 1101 (500-2250) | 2100 (1000-4000) | <.001 |
| Diuretics | 877 (21.6) | 701 (35.6) | <.001 |

**Abbreviations:** ACS, acute coronary syndrome; AKI, acute kidney injury; APACHE III, Acute Physiology and Chronic Health Evaluation score; CHF, congestive heart failure; COPD, chronic obstructive pulmonary disease; CVA, cerebrovascular accident; CCI, Charlson comorbidity index; MV, mechanical ventilation; ICU, intensive care unit; PVD, peripheral vascular disease; Scr, serum creatinine; SOFA, Sequential Organ Failure Assessment score.

^a^ Continuous variables are expressed as mean (±SD) or median and interquartile range (IQR); categorical variables are expressed as frequency (n) and percentage (%).
