# Supplementary material for: Dyschloremia Is a Risk Factor for the Development of Acute Kidney Injury in Critically Ill Patients
Source: PLoS One. 2016 Aug 4;11(8):e0160322. doi: 10.1371/journal.pone.0160322 (PMC4974002; doi:10.1371/journal.pone.0160322)
Supplement: S2 Table — (DOCX) [file pone.0160322.s002.docx]

**Supplementary Table 2.** Outcome of Hyperchloremic Patients with or without Acidemia *

| Variable | Hyperchloremia | | |
| --- | --- | --- | --- |
|  | pH <7.30  (N=135) | pH ≥7.30  (N=122) | *P* value |
| **ICU admission, n (%)** |  |  | 0.9 |
| CCU &MICU | 102 (76.0) | 91(74.6) |  |
| SICU | 33(24.0) | 31(25.4) |  |
| **Demographic characteristics** | | | |
| Age*,* years | 58(41-79) | 60. (40.-78) | 0.9 |
| Male | 66(48.9) | 68(55.7) | 0.3 |
| **Score** | | | |
| Charlson score | 1(0-4) | 1(0-3) | 0.03 |
| APACHEIII | 63(43-79) | 53(37-78) | 0.2 |
| SOFA | 4(2-6) | 3(2-7) | 0.4 |
| **Outcomes** | | | |
| AKI | 45 (33.3) | 43 (35.3) | 0.7 |
| AKI stage I | 25 (18.5) | 25 (20.5) | 0.7 |
| AKI stage II | 6 (4.4) | 8 (6.6) | 0.5 |
| AKI stage III | 14 (10.4) | 10 (8.2) | 0.5 |
| Use of MV | 52(38.5) | 35(28.7) | 0.09 |
| ICU LOS, days | 1.2(0.8-2.4) | 1.1(0.9-1.9) | 0.2 |
| ICU mortality, n (%) | 6(4.4) | 5(4.1) | 0.9 |
| Hospital LOS, days | 3.9(1.9-7.6) | 3.9(1.9-8.1) | 0.6 |
| Hospital mortality, n (%) | 8(5.9) | 10(8.2) | 0.5 |
| **Lab test** | | | |
| **Baseline Scr** | **N=133** | **N=121** |  |
| Baseline Scr, mg/dL | 1.1(0.8-1.5) | 1.1(0.7-1.4) | 0.2 |
| **Baseline albumin** | **N=23** | **N=23** |  |
| Baseline albumin, g/dL | 3.2(2.7-3.7) | 3.3(2.7-3.8) | 0.9 |
| Baseline Chloride, (mmol/L) | 111(110-114) | 110(109-111) | <.0001 |
| Baseline Sodium (mmol/L) | 142 (139-144) | 141(139-143) | 0.01 |
| pH | 7.25.(7.22-7.27) | 7.35(7.32-7.38) | <.0001 |
| **Fluids and diuretics** | **N=123** | **N=116** |  |
| 0.9% Saline (mL) | 1450(600-3491) | 1275(1000-2427) | 0.2 |
| Diuretic use (%) | 6(4) | 11(9) | 0.1 |

**Abbreviations:** AKI, acute kidney injury; APACHE III, Acute Physiology and Chronic Health Evaluation score; MV, mechanical ventilation; ICU, intensive care unit; Scr, serum creatinine; SOFA, Sequential Organ Failure Assessment score

*Continuous variables are expressed as mean (±SD) or median and interquartile range (IQR); categorical variables are expressed as frequency (n) and percentage (%).
