# Supplementary material for: Dyschloremia Is a Risk Factor for the Development of Acute Kidney Injury in Critically Ill Patients
Source: PLoS One. 2016 Aug 4;11(8):e0160322. doi: 10.1371/journal.pone.0160322 (PMC4974002; doi:10.1371/journal.pone.0160322)
Supplement: S3 Table — (DOCX) [file pone.0160322.s003.docx]

**Supplementary Table 3.** Multivariable Analysis of In-Hospital Mortality

|  | **Univariate Analysis** | | **Multivariate Analysis** | | |
| --- | --- | --- | --- | --- | --- |
| **Variable** | **OR; 95% CI** | ***P-*val** | **OR; 95% CI** | ***P-*val** |  |
| Age, year | 1.04 (1.04-1.05) | <.001 | 1.04 (1.03-1.05) | <.001 |  |
| Male | 0.86 (0.71-1.05) | .1 | 0.92 (0.71-1.19) | .5 |  |
| SOFA Score | 1.44 (1.40-1.49) | <.001 | 1.46 (1.21-1.41) | <.001 |  |
| Charlson comorbidity index | 1.18 (1.14-1.21) | <.001 | 1.10 (1.05-1.14) | <.001 |  |
| Baseline chloride, ≤94 mmol/L | 2.07 (1.58-2.68) | <.001 | 2.13 (1.52-3.00) | <.001 |  |
| Baseline chloride, 94-100 mmol/L | 1.14 (0.89-1.45) | .3 | 1.27 (0.93-1.74) | .1 |  |
| Baseline chloride,100-108 mmol/L | 1.00 (reference) | -- | 1.00 (reference) | -- |  |
| Baseline chloride, >108 mmol/L | 1.13 (0.71-1.91) | .6 | 1.01 (0.54-1.79) | .9 |  |

**Abbreviations:** SOFA, Sequential Organ Failure Assessment score
